# Bladder Metastasis from Breast Cancer: A Systematic Review

**DOI:** 10.7759/cureus.7408

**Published:** 2020-03-25

**Authors:** Uday Karjol, Pavan Jonnada, Sushma Cherukuru, Ajay Chandranath

**Affiliations:** 1 Surgical Oncology, Kidwai Memorial Institute of Oncology, Bangalore, IND; 2 Pathology, AMPATH Laboratories, Hyderabad, IND

**Keywords:** bladder metastasis, breast cancer, oncology, breast neoplasm, urinary bladder neoplasms, distant metastasis

## Abstract

Breast cancer is the leading cause of cancer death in women, and most breast cancer related deaths are due to metastasis. Urinary bladder metastasis from breast cancer is rarely reported in the literature. In this review, we examined the reported cases of breast cancer metastasizing to the urinary bladder, with the objective of identifying clues that could help physicians in diagnosing and planning further treatment. We performed a systematic review of the literature to analyze the clinical and pathological profile of this disease. We thoroughly examined and systematically reported data regarding epidemiology, the pattern of spread, signs and symptoms, pathology and hormonal status, diagnostic workup, management, and outcomes. Urinary bladder metastases from breast cancers are more common in invasive lobular carcinoma. In addition to asymptomatic presentations, most cases present with hematuria and voiding dysfunction. This review summarizes the insights into the incidence, clinical presentation, diagnostic workup, management, and prognosis of urinary bladder metastasis in patients with breast cancer.

## Introduction and background

Breast cancer is the most common cancer, accounting for almost one in four cancer cases among women [1]. The incidence of breast cancer is highest in Australia, Europe, and North America. Mortality due to breast cancer is highest in Fiji [1]. Despite increased awareness, screening programs, and advancements in treatment, breast cancer remains the leading cause of cancer death in women. Most breast cancer related deaths are due to metastasis. Urinary bladder metastasis from breast cancer is a real phenomenon but rarely reported in the literature.

**Figure 1:**
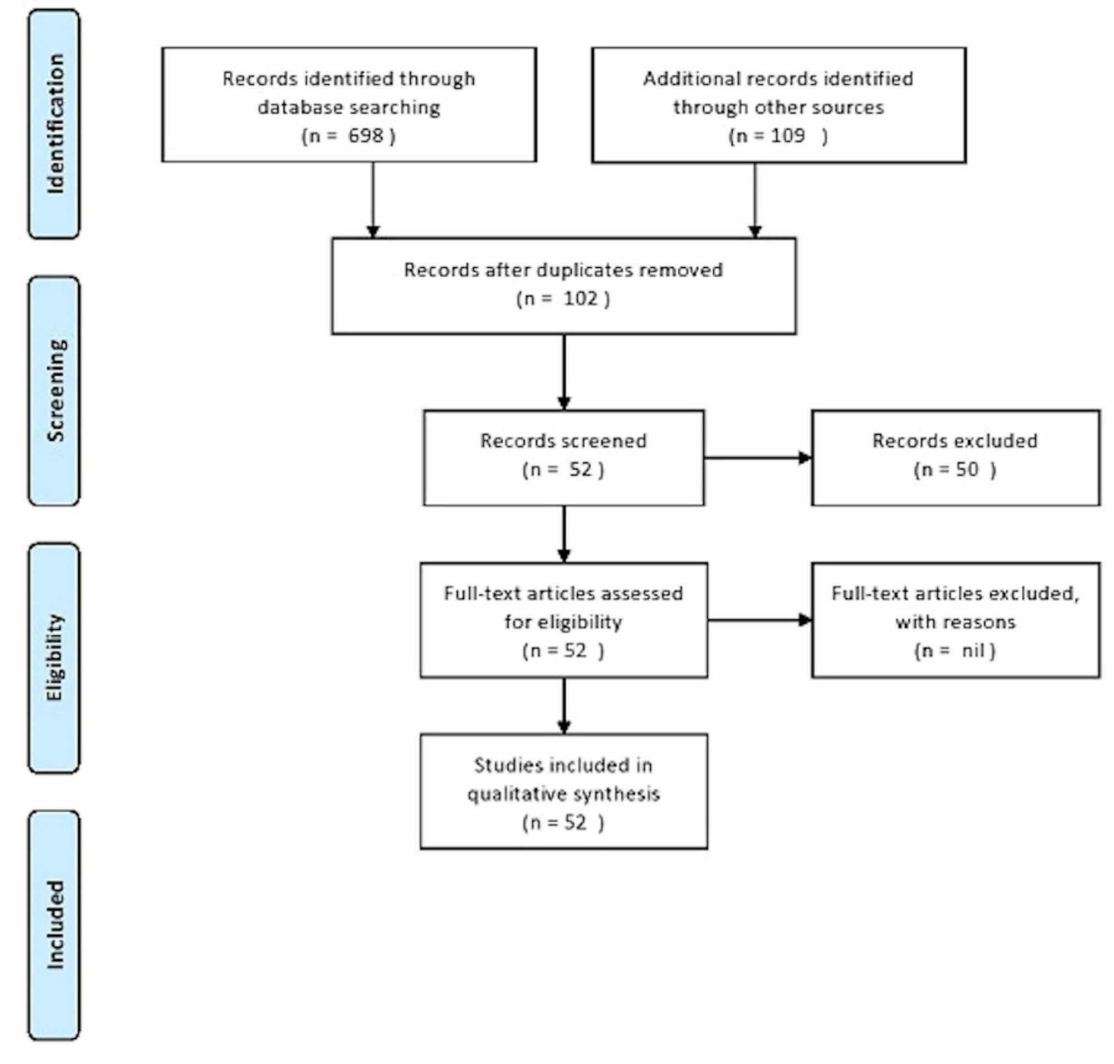
PRISMA flow chart PRISMA, Preferred Reporting Items for Systematic Reviews and Meta-Analyses

In this review, we examined the reported cases of breast cancer metastasizing to the urinary bladder to identify clues that could help physicians in diagnosing and planning further treatment. We electronically searched PubMed, Medline, and Google Scholar databases for articles published between 1950 and 2019 using the following keywords: “bladder metastasis”, “breast cancer”, “oncology”, “breast neoplasm”, “urinary bladder neoplasms”, and “distant metastasis”. All case reports, case series, and review articles were thoroughly examined, and data regarding epidemiology, the pattern of spread, signs and symptoms, pathology and hormonal status, diagnostic workup, management, and outcomes were systematically reported. The PRISMA (Preferred Reporting Items for Systematic Reviews and Meta-Analyses) flow chart is shown in Figure 1.

## Review

Though rare, secondary tumors of the urinary bladder can present a diagnostic dilemma in considering a differential diagnosis of primary bladder cancer. They comprise less than 2% of all bladder cancers [2]. The most common primary sources are the stomach, breast, colon cancer, and melanoma [2]. Most of these secondary tumors are because of direct extension from another pelvic neoplasm, such as sigmoid, prostate, or cervical cancer. Metastases from distant organs are sporadically reported in the literature, with the most common being the stomach, lung, and melanoma [3].

Approximately 45% of breast cancers present with metastasis, which can occur in almost any organ. Breast cancer frequently metastasizes to the bone, lung, liver, and brain, known as organ tropism [4]. For patients with metastatic breast cancer (MBC), 30% to 60% have lesions in the bone, 4% to 10% in the brain, 15% to 32% in the liver, and 21% to 32% in the lung [5]. Urinary bladder metastases (UBMs) are rare and reported in the literature occasionally.

UBMs from breast cancer are extremely rare, with about 65 cases reported in the literature. Autopsy studies analyzing UBM from breast cancer reported the incidence ranging from none to almost 7%. In 1950, in a monumental analysis of 1,000 autopsies, Abrams et al. identified four (2%) cases of UBM after reviewing 167 cases of MBC [6]. In 1951, Klinger identified three (<1%) cases of UBM from MBC after reviewing 5,000 autopsy cases of genitourinary tract metastases [7]. Ganem and Batal surveyed the published autopsy reports through 1956 and found 16 cases of UBM [8]. In 1967, Goldstein identified four cases of UBM (1.2%) from MBC after reviewing 341 autopsy cases of patients who died of advanced breast cancer [9]. Pontes and Oldford reported approximately 7% of UBM among 85 autopsies [10].

The pattern of metastasis of breast cancer may be related to the histological type of cancer [11]. The major pathological subtypes of breast cancer are ductal and lobular carcinoma. Invasive ductal carcinoma (IDC) is the most common subtype of breast cancer, accounting for 90% of cases [12]. Invasive lobular carcinoma (ILC) is the second most common subtype, accounting for 8% to 14% of cases [13]. UBMs from breast cancer are more common in ILC than IDC. The metastatic pattern of ILC differs from IDC in that it tends to occur as a diffuse thickening of mucosa rather than a discrete nodule. It has a higher propensity to metastasize to serosal surfaces (gynecological and gastrointestinal tracts), and the spread from these sites may be the cause for the greater incidence of bladder involvement [11,14,15]. Feldman et al. noted that one-third of patients with UBM had ILC [11]. Borst and Ingold noted that 3% of ILC metastasized to peritoneum-retroperitoneum compared with 0.6% of IDC [13].

The pattern of spread to UBM from breast cancer is because of tumor embolus that does not seed in the lung but passes through pulmonary circulation and reaches the target organ and soil there, causing a metastasis [8-10]. Pontes and Oldford postulated that breast cancer metastasizes to the bladder through retroperitoneal involvement [10]. Patients receiving steroids may develop metastasis at unusual sites due to the possible influence of the immunosuppressive effect of steroids [16].

Based on gene expression profiles and receptor status (estrogen receptor [ER], progesterone receptor [PR], human epidermal growth factor receptor 2 [HER2]) and on proliferation status as assessed by Ki67, Perou et al. subdivided breast cancer into four main clinical subtypes [17]: luminal A (ER+/PR+), luminal B (ER+/PR+/HER2−/+/Ki67+), HER2 overexpressing (ER−/PR−/HER+), and basal-like/triple-negative (TN) (ER−/PR−/HER2−). The expression of ER and PR in the UBM is expected to be similar to that in primary breast cancer. However, discordant expression of the ER and PR status in primary breast cancer and metastatic bladder lesions have been observed in studies [11,18,19]. Iguchi et al. hypothesized that the heterogenous expression of ER and PR between the primary tumor and metastatic lesion is based on cell clonality; breast cancer cells are polygonal. Besides clonal theory, change of expression can occur after adjuvant therapy due to the elimination and growth of PR-positive or ER-negative cells or due to gene mutations [20].

Synchronous and metachronous presentations are observed for UBM from breast cancer [21,22]. The majority of UBMs present as a part of widespread disease. Solitary metastasis to the urinary bladder without evidence of any other distant disease has been sporadically reported [11,14,19,23]. Clinical presentation can range from asymptomatic presentation to gross hematuria, obstructive uropathy, and renal failure [11,19,24,25]. The most common presenting symptom is painless gross hematuria. Gross hematuria with a history of breast cancer should be carefully investigated, considering the side effects of cyclophosphamide as a treatment of primary breast cancer, regardless of time or duration of treatment. Feldman et al. reported in their study that 17 of 19 patients reviewed presented with urinary symptoms of frequency, nocturia, incontinence, dysuria, microscopic hematuria, and back pain [11]. Detrusor involvement can lead to irritative voiding symptoms and can present earlier than hematuria. Balachandran and Duckett reported in their study that their patient presented with urgency and nocturia and that urodynamic testing revealed overactivity of the detrusor [26]. Soon et al. reported that UBMs were detected before the diagnosis of breast cancer and concluded that urinary incontinence might be the first sign of cancer; therefore, a careful evaluation for the possibility of UBM should be sought in patients with a history of breast cancer and urinary symptoms [21]. Focused ultrasonography of the urinary bladder is the first-line diagnostic investigation for a patient of breast cancer with voiding symptoms. The critical diagnostic modalities during workup for suspicious UBM are cystoscopy and biopsy. Cystoscopy is useful in identifying the disease and can aid resection. However, random biopsies taken in standard cystoscopy can reveal UBM [11,26,27]. Contrast-enhanced CT becomes valuable when there is evidence of renal impairment, suggesting obstructive nephropathy or equivocal cystoscopy [28]. Fluorodeoxyglucose positron emission tomography can improve the prediction of the clinical outcome of previously treated patients and improve the imaging evaluation of bladder neoplasm [29].

In our opinion, due to varied presentations of UBM from breast cancer, we suggest that biopsies should be undertaken in a patient suggestive of UBM from breast cancer or having symptoms. This is because of a missed examination of breast masses or the absence of early diagnosis programs.

UBM from breast cancer may present from one month to 30 years after the initial diagnosis of the primary tumor. Simultaneous presentation of UBM and breast cancer was reported [3,24,30]. It is worth mentioning that rarely the diagnosis of breast cancer followed the diagnosis of UBM [11]. The mean time of UBM from the primary diagnosis of breast cancer is 90 months, as reported in a large series [11]. Following the diagnosis of UBM, transurethral resection of the bladder lesion should be undertaken to stop hematuria. This also facilitates the stenting of ureters in the case of ureter obstruction. Percutaneous nephrostomy can also be undertaken to normalize renal function [25].

A combination of endocrine therapy and chemotherapy is the mainstay of treatment for UBM from breast cancer. The response to endocrine therapy is superior in the luminal A group of patients when compared with other subgroups and prolongs the disease-free survival in the luminal A group [23]. The prognosis of MBC to urinary bladder is similar to that of any MBC. The average survival is 18 to 30 months [31]. However, there are reports stating UBM has a worse prognosis than bone metastasis [32]. The reason postulated is that symptomatic UBM from breast cancer is detected at a late stage. The mucosal breach leads to gross hematuria, leading to investigations [33]. The majority of UBMs from breast cancer presented as part of systemic disease, which impacts survival. The reports stated that solitary UBM had shown increased disease-free survival after treatment [11,14,19,23]. The role of radiotherapy is its ability to control hematuria and local control of the disease [34].

## Conclusions

UBMs from breast cancer are rare, yet there is an increase in reports of such metastasis over the last few years, owing to better imaging and diagnostic modalities. The majority of UBMs present as part of widespread metastatic disease. Most UBMs occur in invasive lobular cancer. In addition to asymptomatic presentations, most cases present with hematuria and voiding dysfunction. After diagnosis with imaging, cystoscopy should be part of the workup in the biopsy of the lesion and performing transurethral resection. If no lesion is seen in a suspected case of UBM from breast cancer, random biopsies should be warranted. Chemotherapy and hormonal therapy are the mainstays of management, with radiotherapy being used to control bleeding. The prognosis of UBM is poor unless UBM represents the only metastatic site. Hence, patients with a history of breast cancer and urinary symptoms should be thoroughly evaluated for bladder metastasis.
